# Cryptorchidism and Infertility in Rats with Targeted Disruption of the *Adamts16* Locus

**DOI:** 10.1371/journal.pone.0100967

**Published:** 2014-07-01

**Authors:** Shakila Abdul-Majeed, Blair Mell, Surya M. Nauli, Bina Joe

**Affiliations:** 1 Center for Hypertension and Personalized Medicine, Program in Physiological Genomics, Department of Physiology and Pharmacology, University of Toledo College of Medicine and Life Sciences, Toledo, Ohio, United States of America; 2 Department of Pharmacology, University of Toledo College of Pharmacy, Toledo, Ohio, United States of America; National Cancer Institute, United States of America

## Abstract

A Disintegrin And Metalloproteinase with ThromboSpondin motifs16 (ADAMTS-16) is a member of a family of metalloproteinases. Using a novel zinc-finger nuclease based gene-edited rat model harboring a targeted mutation of the *Adamts16* locus, we previously reported this gene to be linked to blood pressure regulation. Here we document our observation with this model that *Adamts16* is essential for normal development of the testis. Absence of *Adamts16* in the homozygous *Adamts16^mutant^* males resulted in cryptorchidism and male sterility. Heterozygous *Adamts16^mutant^* males were normal, indicating that this is a recessive trait. Testes of homozygous *Adamts16^mutant^* males were significantly smaller with significant histological changes associated with the lack of sperm production. Temporal histological assessments of the testis demonstrated that the seminiferous tubules did not support active spermatogenesis, but progressively lost germ cells, accumulated vacuoles and did not have any sperm. These observations, taken together with our previous report of renal abnormalities observed with the same *Adamts16^mutant^* rats, suggest an important mechanistic link between *Adamts16* and the functioning of the male genitourinary system.

## Introduction

ADAMTS (A Disintegrin And Metalloproteinase with ThromboSpondin motifs) are a family of zinc-binding proteinases with multiple domains and varying numbers of thrombospondin like motifs [1], [2]. ADAMTS-16 is a member of the ADAMTS family. Expression of Adamts16 has been reported in rodent fetal lung and kidneys, adult brain and ovaries [3], [4], and most recently in mice embryonic and adult rat gonads [5]. Several studies have implicated ADAMTS16 in various pathological conditions, including osteoarthritis [6], [7], colorectal and esophageal cancers [8], hypertension [9], [10] and physical functional impairment in the context of major mental illnesses including bipolar disorder, major depressive disorder and schizophrenia [11]. A recent study reports that murine Adamts16 is co-expressed with Wilms tumor protein, Wt1, in embryonic gonads and adult testes and spermatids [5].

We recently generated a null *Adamts16^mutant^* rat model by deleting 17 base pairs of the first exon of *Adamts16* from the genome of the Dahl salt-sensitive (S) rat using the zinc-finger nuclease technology [9]. Both, the homozygous *Adamts16^mutant^* as well as the heterozygous model *Adamts16^mutant^* exhibited significantly low systolic and diastolic blood pressures compared to the salt-sensitive Dahl-S rat [9]. One unexpected morphological observation as a result of this genetic modification of the Dahl-S rat was that the *Adamts16^mutant^* homozygous male rats demonstrated cryptorchidism and were infertile. The current report details this observation, which, when taken together with previous observations of renal abnormalities observed in the same rats, suggests that *Adamts16* is a critical gene required for the normal functioning of the genitourinary system.

## Methods

### Experimental Animals

All animal research protocols were preapproved by the University of Toledo Animal Care and Use Committee and conducted as per the Guide for the Care and Use of Animals of the National Institutes of Health. The Dahl Salt–sensitive (S) rats were from our inbred colony maintained at the University of Toledo Health Science Campus. *Adamts16^Mutant^* rats, which are S rats with targeted disruption of the *Adamts16* locus, were generated using the zinc-finger nuclease method as detailed elsewhere [9].

### Histology

Testes were harvested and processed from concomitantly raised S rats, homozygous *Adamts16^Mutant^* rats and heterozygous *Adamts16^Mutant^* rats. Animals were euthanized using CO_2_, and tissues were transcardially perfused with 4% PFA. Testes were excised, weighed and immediately transferred to the prepared plastic moulds in dry ice or to pre-labeled 50 mL falcon tubes containing 30–35 mL Bouin's fixative. Tissues were processed simultaneously for frozen OCT (optimum cutting temperature) technique as well as for paraffin-embedding.

OCT technique: Partially filled plastic molds with OCT were labeled and kept in dry ice before euthanizing the animals. After tissue harvest, additional OCT was added and the tissues frozen to −80°C. Tissues were thawed to −20°C and 10 µm sections were obtained using the Leica cryostat apparatus maintained at −20°C. Sections were stored at −80°C until further use.

Paraffin Embedding: Testes samples were maintained in Bouin's fixative for 6–8 hours at room temperature. The tissues were then transferred to fresh Bouin's fixative for 24 hours, dehydrated using graded ethyl alcohol and embedded in paraffin. Sections (5 µm) were prepared and stored at room temperature until further use.

### Periodic acid- Schiff's Reagent-Hematoxylin (PSH) staining

Paraffin embedded tissues of all age groups of all the three phenotypes were processed simultaneously to maintain identical experimental conditions. Slides were deparaffinized using histoclear, rehydrated using graded ethyl alcohol followed by dH_2_O and incubated with 1% periodic acid for 20 minutes, washed and incubated in Schiff's reagent for 30 minutes at room temperature. The slides were counter stained using Harris hematoxylin, dehydrated using graded ethyl alcohol, 100% xylene and stored using perma mount and cover slip.

### Immuno- staining for primary cilia and proliferating cell nuclear antigen (PCNA)

Paraffin embedded tissues from 50day old rats were used to study primary cilia and proliferating cell nuclear antigen (PCNA) were observed using fluorescence microscopy. Primary cilia was stained using acetylated α-tubulin (Sigma clone 6-11B-1) 1∶500 dilution overnight at 4°C and anti-PCNA (Santa Cruz, (C20) # sc-9857-R) 1∶300 dilution overnight at 4°C. The slides were initially de-paraffinized using histoclear, rehydrated in graded ethyl alcohol and PBS. After antigen retrieval with sodium citrate, the slides were washed with 0.1M glycine at room temperature, followed by incubation in ice cold 0.1% sodium borohydride in ice cold HBSS for 40 minutes. The slides were first stained for cilia using anti-mouse acetylated α-tubulin and then with anti-rabbit PCNA antibody. Nuclei were stained using mounting media containing DAPI. Images were visualized and captured with an inverted Nikon Ti-U microscope.

### TUNEL Assay

Rat testis from 50 day old animals were collected and fixed in 10% natural buffered formalin (NBF), embedded in paraffin, and sectioned at 5 µm thickness. Apoptotic cells in the seminiferous tubules were stained by the TUNEL method using an ApopTag Fluorescein In Situ Apoptosis Detection kit (green, catalog no. S7110; Millipore). Nuclei were visualized by DAPI staining (blue, catalog no. H-1500; Vector Labs). Images were visualized at 10X magnification and captured with an inverted Nikon TE2000-U.

### *In-situ* hybridization

Locked nucleic acid (LNA) modified DNA oligonucleotide probes labeled at the 5′ and 3′ ends with DIG were supplied by Exiqon, Inc. The LNA probe was custom designed to hybridize with the following target sequence of rat *Adamts16*: TTTGC*gctctgggtgctgttgc*TGGCG. The sequence shown in italics is the 17 bp sequence, which is intact in the wild-type S rats, but absent in the *Adamts16^mutant^* rats [9]. Frozen tissue sections from 30 day old animals were used for the *in situ* experiment conducted as per a previously published protocol [12].

## Results

### Anatomy and morphology of *Adamts16^mutant^* rat testes

**Figure 1:**
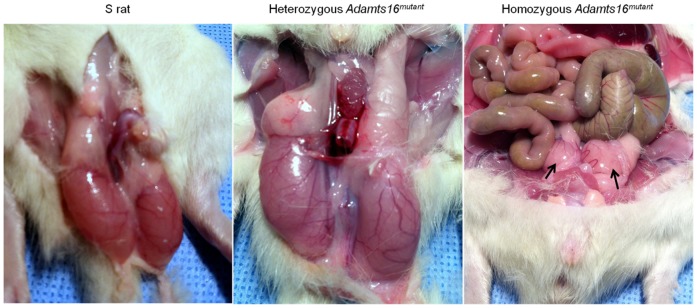
Cryptorchidism in homozygous *Adamts16^mutant^* rats. Representative images exposing rat testes from age-matched 50 day old rats. Black arrows point to smaller, undescended testes in homozygous *Adamts16^mutant^* rats.

**Figure 2:**
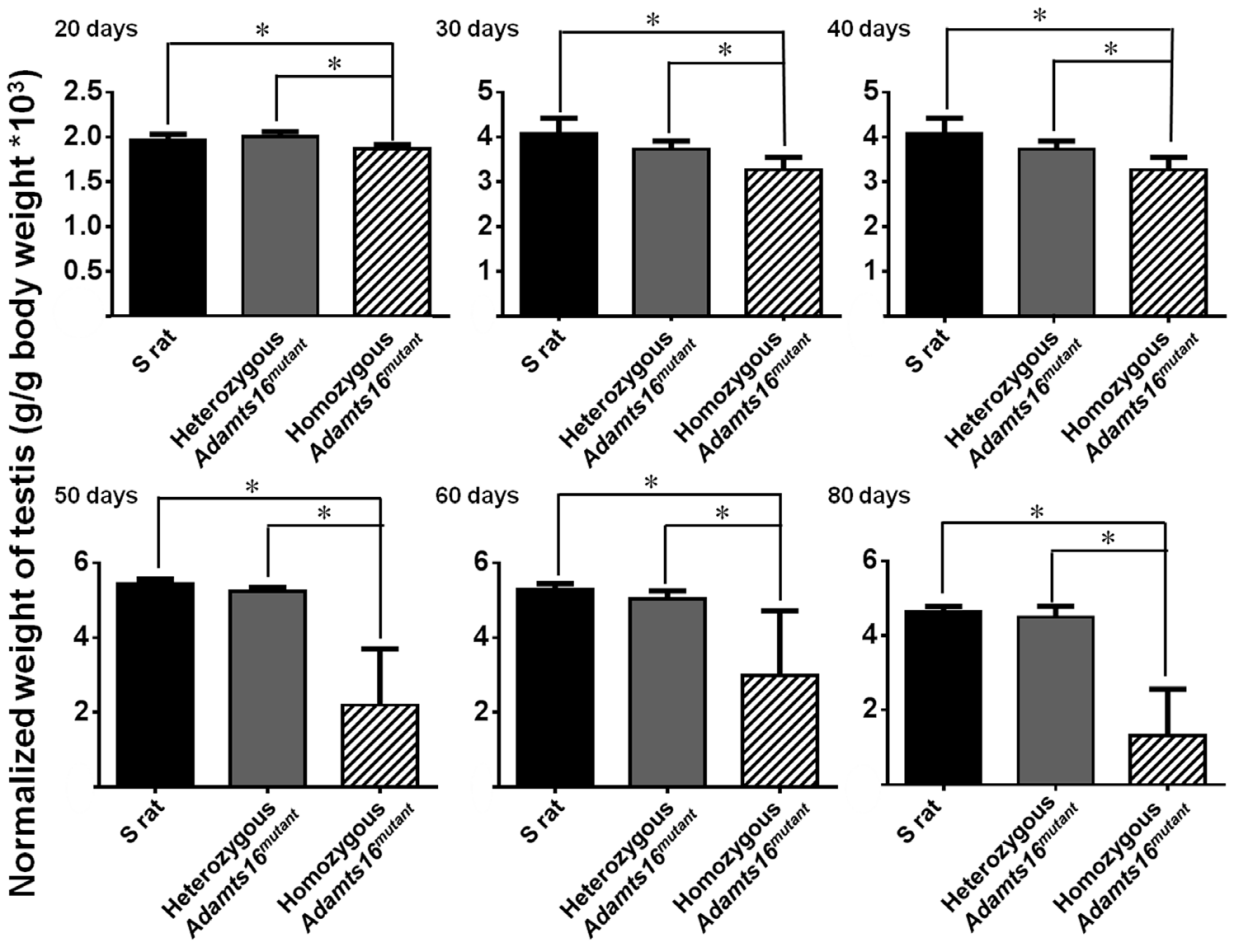
Comparisons of normalized testes weights. Bars represent weights of testes from age-matched rats (n = 3/group at each time point) normalized to their body weights. *p<0.05.

Homozygous and heterozygous *Adamts16^mutant^* males were observed and compared with S rats for at least 4 generations. Through all the generations, the homozygous *Adamts16^mutant^* male rats exhibited bilateral cryptorchidism (Figure 1). When paired with either female homozygous or heterozygous *Adamts16^mutant^* rats or with female S rats, these *Adamts16^mutant^* male rats did not generate pups. However, homozygous *Adamts16^mutant^* female rats bred with heterozygous *Adamts16^mutant^* male rats resulted in litters. Thus we concluded that homozygous *Adamts16^mutant^* male, but not female rats, were sterile. Testes from 20 to 80 day old homozygous and heterozygous *Adamts16^mutant^* males were morphologically compared with age-matched Dahl S rats (Figures 1 and 2). Testes from all the age groups of rats studied were significantly smaller in size and weighed lesser from the homozygous *Adamts16^mutant^* males compared to the heterozygous *Adamts16^mutant^* males and S rats (Figure 2). The differences between the groups were more pronounced as the animals advanced in age (Figure 2).

### Localization of Adamts16 within the rat testes

**Figure 3:**
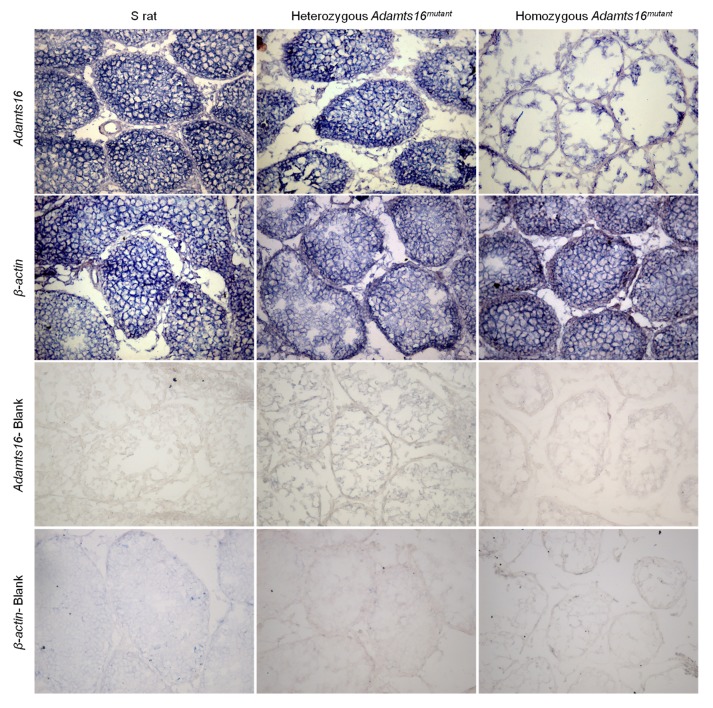
Detection of *Adamts16* mRNA within the rat testes. Presence of *Adamts16* mRNA in the testes of age-matched 30 day old animals was determined by *in-situ* hybridization as described under the methods section. *β-actin* was used as a control.

*Adamts16* mRNA was visualized in the testes sections of 30 day old animals by *in-situ* hybridization. Staining for *Adamts16* mRNA was observed in the testes sections from the S rats as well as the *Adamts16^mutant^* heterozygous testes. Staining in the *Adamts16^mutant^* homozygotes was very weak, potentially due to complementarity of the LNA probe immediately flanking the 17bp deletion to the *Adamts16^mutant^* mRNA. Staining for Adamts16 was observed throughout the interstitial tissue surrounding the seminiferous tubule and adjoining basement membrane/parenchyma (Figure 3). The pattern of localization was similar to that of β-actin (Figure 3).

### Testicular histological abnormalities of homozygous *Adamts16^mutant^* rats

**Figure 4:**
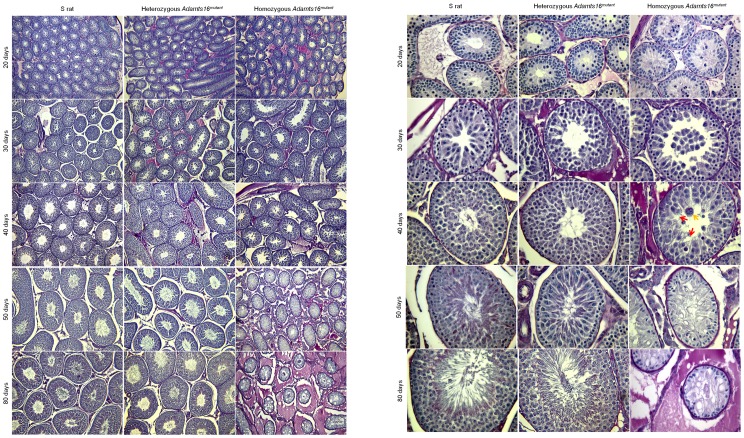
Temporal histological changes in rat testes. Testes from n = 3 animals each from the various groups of animals were observed under (a) 10X and (b) 40X magnification post PSH staining as described under the methods section. Representative images are shown.

Temporal histological changes were assessed using testicular sections of S rats, and heterozygous and homozygous *Adamts16^mutant^* rats. As evident from Figure 4a, the dense clustering of the seminiferous tubules seen at all the time points assessed was comparable between the S rats and the heterozygous *Adamts16^mutant^* rats. However, in the homozygous *Adamts16^mutant^* rats, there was a progressive loss of contact between the seminiferous tubules associated with shrinkage of the sizes of the seminiferous tubules. By 50 days of age, the testis section from homozygous *Adamts16^mutant^* rats were histologically distinct with the seminiferous tubules widely separated from each other by interstitial tissue and by 80 days, there was significant accumulation of densely stained interstitial tissues and reticular fibers (Fig 4a and 4b, pink color in 80 days, homozygous *Adamts16^mutant^* rats).

**Figure 5:**
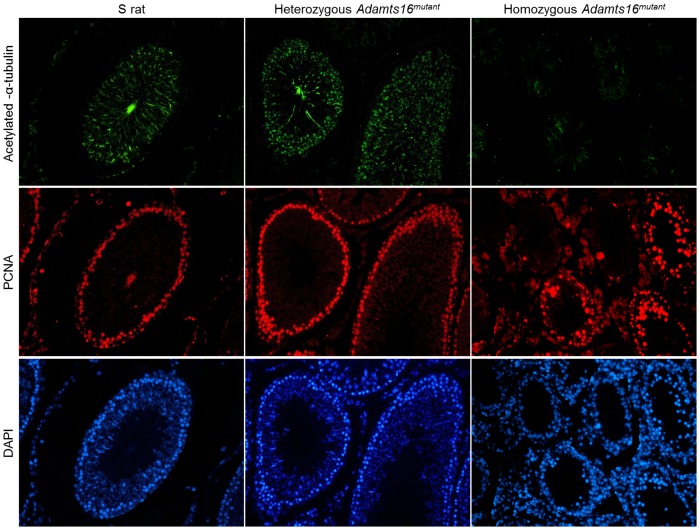
Immuno-histochemical staining for cilia and proliferating cells within the seminiferous tubules. Fifty-day old age-matched rat testis sections were immunostained for cilia using α-tubulin (green), proliferating cells with PCNA (red) and nuclei (blue).

**Figure 6:**
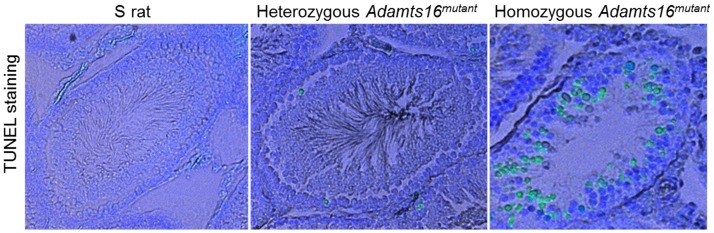
TUNEL staining for apoptosis and images of sperms within the seminiferous tubules. Fifty-day old age-matched rat testis sections were assessed for apoptosis as described under the methods section. Apoptotic cells are stained green.

**Figure 7:**
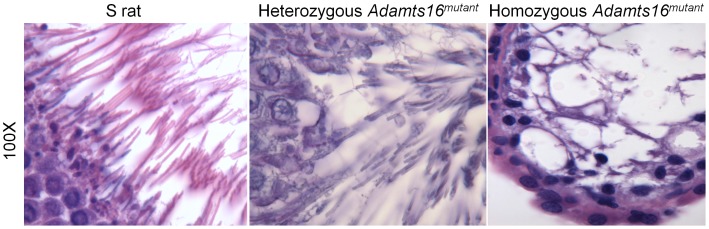
Visualization of sperms in the seminiferous tubules Sixty-day old age-matched rat testis sections from 3 rats/group were visualized under a 100X microscope after PSH staining as described under the methods section. Representative images are shown. Elongates structures seen in the fields of S and heterozygous *Adamts16^mutant^* rats are sperms. These structures are not seen in the homozygous *Adamts16^mutant^* rats.

Histological examination of the seminiferous tubules at a larger, 40X magnification are provided in Figure 4b. The general organization of the spermatogonial cells around a distinct lumen as seen in the S rats and in the heterozygous *Adamts16^mutant^* rats was distorted in the homozygous *Adamts16^mutant^* rats. The characteristic lumen was absent in the homozygous animals (Fig 4b, 20 days, homozygous *Adamts16^mutant^* rats). The lumen was filled with what appears to be multinucleated giant cells and vacuoles. (Figure 4b, 30 and 40 days, homozygous *Adamts16^mutant^* rats). At the 40 day time point (Figure 4b), which is when spermatogenesis begins, the seminiferous tubules from S and heterozygous *Adamts16^mutant^* rats were comparable and filled with expanding numbers of cells within the tubules and flagellated sperms appearing within the lumen whereas the seminiferous tubules of the homozygous *Adamts16^mutant^* rats were with fewer cells and with grossly enlarged, giant nucleated cells and vacuoles. By 50 days, a time point when the rats are sexually mature, there was a total collapse of structural organization characterized by cell depletion by the epithelial lining and lack of lumen (Figure 4a and b). At this stage, as observed by PCNA staining (Figure 5), dividing cells were almost completely absent in the seminiferous tubules from the homozygous *Adamts16^mutant^* rats. Also, DAPI staining of the seminiferous tubules from the homozygous *Adamts16^mutant^* rats suggested loss of nuclei, indicating enhanced cell death within the shrunken seminiferous tubules (Figure 5). Cellular Apoptosis was further confirmed by TUNEL assay (Figure 6). Apoptotic cells were observed within the seminiferous tubules from the homozygous Adamts16*^mutant^* rats alone but not within the seminiferous tubules of either the heterozygous Adamts16*^mutant^* rats or the S rats.w (Figure 6). Further, the homozygous *Adamts16^mutant^* rats lacked flagellated sperms as confirmed by staining for motile cilia using acetylated α-tubulin (Figure 5). These observations were further confirmed with images obtained at higher magnification that clearly indicated the lack of sperms in the *Adamts16^mutant^* rats (Figure 7).

## Discussion

ADAMTS-16 belongs to the Adamts family of mammalian metalloproteinases. Emerging studies in recent years document the physiological importance of Adamts16 in a variety of organ systems. Using a targeted gene-disruption rat model of *Adamts16*, we previously validated a genetic link between the *Adamts16* locus and hypertension [9], [10]. Specifically, a 17-bp deletion in the first exon of the *Adamts16* locus lowered blood pressure of a genetically hypertensive rat. In the current study, we report an unexpected consequence of this deletion on the male reproductive system. Homozygous *Adamts16^mutant^* male rats exhibited bilateral cryptorchidism and were infertile. Infertility was associated with the absence of *Adamts16* transcripts in the testis, significantly diminished size of the testis, an age-dependent attrition of the seminiferous tubule and lack of spermatogenesis.

Cryptorchidism or the inability of the testes to descend to the scrotum is mainly associated with infertility [13]. Male fertility is greatly dependent on a timely descent of the testis to the scrotum, and the formation of the two main compartments of the testis; the interstitium and the seminiferous tubules essential for spermatogenesis. Testicular descent to the scrotum is enabled by the gubernaculum, which steers the testis to, and anchors it to the scrotum. The embryonic gubernaculum in rodents is a mesenchymal structure composed of mesenchymal cells and loose extracellular matrix, including fibroblasts and smooth muscle cells, located cranially to the intra-abdominal cone. After birth, the gubernaculum undergoes extensive remodeling of the extra cellular matrix (ECM), elongates to form a subcutaneous sack, and everts its position caudally to enable the testes to be anchored to the scrotum; followed by cyclic remodeling of the seminiferous tubules. With the help of the Sertoli cells, the germ cells develop, move towards the tubular lumen, cross the blood-testis barrier, undergo meiosis and are finally extruded into the lumen as sperm cells. The entire process of spermatogenesis needs to occur without disturbing the integrity of the seminiferous tubule [14]–[17]. Both these processes, maturation of the gubernaculum and spermatogenesis, involve extensive remodeling of the extra-cellular matrix (ECM) which is achieved by a tight balance of metalloproteinases and their inhibitors. Previous studies have indicated that metalloproteinase MT1-MMP plays an active role in testicular descent and MMP2 plays an important role in spermatogenesis [18]–[20]. Another member of the Adamts family of proteins, Adamts10, has been recently described as being expressed during the later stages of mouse spermatogenesis, incorporated into the acrosomal domain of developing spermatids and suggested to be important for sperm adhesion to the zona pellucida during fertilization [21]. Our study points to Adamts16 as an additional member of the Adamts family of proteins that is necessary for a much earlier phase of normal development of the testis and spermatogenesis. Adamts16 may play an important role in the ECM remodeling that is required for testicular descent, lack of which may be responsible for the observed cryptorchidism and subsequent infertility in homozygous Adamts16^mutant^ male rats. Further studies are required to determine the stage at which testicular descent is arrested in homozygous Adamts16^mutant^ males.

In adult mammalian testes, sertoli and germ cells at different stages of their development in the seminiferous epithelium are in close contact with the basement membrane, a modified form of extracellular matrix (ECM), relying on its structural and hormonal supports [22]. Additionally, the basement membrane is also in close contact with the underlying collagen network and the myoid cell layers, which together with the lymphatic network, constitute the *tunica propria* [22]. The *in situ* data presented, which is obtained before active spermatogenesis occurs in rats (30 day old samples in Figure 4) suggests that *Adamts16* mRNA is not localized to any particular cell type within the seminiferous tubules, but is observed throughout the testicular parenchyma. Histological data from our studies indicate gross abnormalities in the ECM of homozygous *Adamts16^mutant^* rats. At around 50 days, which is when rats are beginning to attain sexual maturity, interstitial hyperplasia is also noted, but between 50–80 days, this is replaced with progressive expansion of testicular parenchyma and notable shrinkage of seminiferous tubules. Given that ADAMTS-16 is a member of a family of proteases and has a metalloproteinase domain, it is tempting to speculate that Adamts16 is a metalloproteinase that cleaves an unidentified protein within the testicular ECM that may be support spermatogenesis to proceed normally within the seminal vesicles. However, besides one report suggesting that Adamts16 may cleave alpha-2 macroglobulin [4], there is no documented evidence affirming Adamts16 as a metalloproteinase. Identification of specific substrate/s of Adamts16 within the testis will be essential to further understand the precise mechanism through which Adamts16 facilitates spermatogenesis. Alternately, similar to ADAMTS10 which interacts with Fibrillin-1 and promotes its deposition in extracellular matrix [23], it is possible that Adamts16 could influence spermatogenesis by interacting with proteins of the ECM.

The genitourinary system is the organ system of the reproductive organs and the urinary system that share a common embryological origin. Genes that function during the development of the genitourinary system are likely to impact the functions of both the gonads and the kidneys. In a previous study with the *Adamts16^mutant^* rats, we observed that despite the lowering of hypertension, these mutant rats had increased renal dysfunction characterized by an increase in proteinuria and significant renal abnormalities [9]. These data, when taken together with the current observation of sterility and gross morphological and anatomical abnormalities associated with lack of spermatogenesis, provides definitive evidence for an important, yet unknown, function of Adamts16 in genitourinary development. Our interpretation is supported by several lines of evidence in the literature. A recent independent study points to Adamts16 as perhaps an important downstream target of a transcription factor, Wilms tumor suppressor protein, Wt1, in both the testes and kidneys [5]. In humans, germline mutations of the WT-1 tumor suppressor gene are associated with urogenital malformations [24]. Also, six missense mutations within *WT1*, were detected in 529 human patients with non-obstructive azoospermia, indicating a strong association between WT1 mutation and non-obstructive azoospermia [25]. *Wt1* mouse knockout studies indicate that *Wt1* is a gene critical for both kidney and gonad development [24]. Given that ADAMTS-16 is now recognized as a downstream target of Wt1, our study suggests that in humans with normal function of the Wt1 protein, functional polymorphisms within its downstream target, the *Adamts16* gene, could alter the function of the ADAMTS-16 protein and thereby independently associate with dysfunction of the genitourinary system.

Finally, contrary to the observation that stimulation of *Adamts16* expression by Wt1 in parietal granulosa cells may have a role in extracellular matrix (ECM) remodeling, which is a prerequisite for oocyte liberation during ovulation [5], in our studies, ovulation appeared to be normal as all the female homozygous *Adamts16^mutant^* rats were fertile and generated normal litters when bred with either heterozygous *Adamts16^mutant^ rats or with S rats*. The reason for this gender-specific disparity in the function of Adamts16 is not known. It is, however, of interest to note that other members of the ADAMTS family are implicated in ECM transformation in the ovary [26]-[30]. The involvement of such other proteases may add functional redundancy, which may facilitate ovulation even in the absence of one or more constituents [31], [32]. Lack of such functional redundancy may be one of the reasons for the observed gender-specific function of Adamts16 in the male reproductive system.
